# miR167d-*ARF*s Module Regulates Flower Opening and Stigma Size in Rice

**DOI:** 10.1186/s12284-022-00587-z

**Published:** 2022-07-25

**Authors:** Zhi-Xue Zhao, Xiao-Xiao Yin, Sha Li, Yu-Ting Peng, Xiu-Lian Yan, Chen Chen, Beenish Hassan, Shi-Xin Zhou, Mei Pu, Jing-Hao Zhao, Xiao-Hong Hu, Guo-Bang Li, He Wang, Ji-Wei Zhang, Yan-Yan Huang, Jing Fan, Yan Li, Wen-Ming Wang

**Affiliations:** State Key Laboratory of Crop Gene Exploration and Utilization in Southwest China, Sichuan Agricultural University, Chengdu, 611130 China

**Keywords:** *Oryza sativa*, miR167d, Auxin response factor, Flower opening, Stigma size

## Abstract

Flower opening and stigma exertion are two critical traits for cross-pollination during seed production of hybrid rice (*Oryza sativa* L.). In this study, we demonstrate that the miR167d-*ARF*s module regulates stigma size and flower opening that is associated with the elongation of stamen filaments and the cell arrangement of lodicules. The overexpression of miR167d (OX167d) resulted in failed elongation of stamen filaments, increased stigma size, and morphological alteration of lodicule, resulting in cleistogamy. Blocking miR167d by target mimicry also led to a morphological alteration of the individual floral organs, including a reduction in stigma size and alteration of lodicule cell morphology, but did not show the cleistogamous phenotype. In addition, the four target genes of miR167d, namely *ARF6*, *ARF12*, *ARF17*, and *ARF25*, have overlapping functions in flower opening and stigma size. The loss-of-function of a single *ARF* gene did not influence the flower opening and stigma size, but *arf12* single mutant showed a reduced plant height and aborted apical spikelets. However, mutation in *ARF12* together with mutation in either *ARF6*, *ARF17*, or *ARF25* led to the same defective phenotypes that were observed in OX167d, including the failed elongation of stamen filaments, increased stigma size, and morphological alteration of lodicule. These findings indicate that the appropriate expression of miR167d is crucial and the miR167d-*ARF*s module plays important roles in the regulation of flower opening and stigma size in rice.

**Supplementary Information:**

The online version contains supplementary material available at 10.1186/s12284-022-00587-z.

## Background

Rice (*Oryza sativa* L.) is one of the most important food crops in the world. The improvement in grain yield has been a major focus of crop breeding programs. The yield of rice grains is determined by four major components, including panicle numbers per plant, spikelet number per panicle, filling rate of the grains, and the grain weight (Sakamoto and Matsuoka 2008). Spikelets and the inner floral organs are crucial to the filling rate of grains and grain weight (Chen et al. 2022). In addition, successful hybrid rice production needs a sufficient number of pollen grains deposited on the stigma lobes of the seed parent (Virmani et al. 2002). The floral traits and flowering behavior traits influence outcrossing in rice (Virmani et al. 2002), including sigma size, style length, stigma exertion, stamen filament length, and flower opening (Virmani et al. 2002). Thus, elucidating the regulatory mechanisms of flower opening and stigma size are important to outcrossing in rice and genetically improve seed production of hybrid rice.

microRNAs (miRNAs), a class of short non-coding RNAs, play essential roles in various biological processes by regulating the expression of their target genes (Zheng and Qu 2015). A variety of miRNAs have been reported to determine various agronomic traits of rice. For example, miR156 targets *SQUAMOSA promoter-binding protein-like transcription factor* (*SPL*) genes that are involved in the regulation of various agronomic traits (Zheng and Qu 2015). Overexpression of miR156 results in rapid leaf/tiller initiation and precocious leaf maturation (Xie et al. 2012). A point mutation of *OsSPL14* at the miR156-target site, which perturbs the cleavage of the *OsSPL14* transcripts by miR156, showed enhanced grain yields (Jiao et al. 2010; Miura et al. 2010). OsmiR397 promotes panicle branching and enlarges grain size, leading to an increase in the grain yield of up to 25% in the field by targeting *OsLAC*, which encodes a laccase-like protein involved in the sensitivity of plants to brassinosteroids (Zhang et al. 2013). OsmiR444a suppresses tillering by negatively regulating *OsMADS57*, which encodes a MADS-domain family transcription factor (Guo et al. 2013). Overexpression of miR172 leads to a decrease in seed weight and floral defects by delaying the transition from spikelet meristem to floral meristem (Zhu et al. 2009). In addition, a coordinated gene network formed by miR172-*APETALA2* (*AP2*) and miR156/miR529-*SPL2* is involved in the regulation of panicle branching and tillering (Wang et al. 2015). The OsmiR396-GROWTH REGULATING FACTOR (GRF) module regulates grain size by directly regulating miR408 (Yang et al. 2021). A novel miR167a-OsARF6-OsAUX3 module regulates grain length and width (Qiao et al. 2021). OsmiR167a-targeted *AUXIN RESPONSE FACTORS* (*ARF*s), namely *OsARF12*, *OsARF17*, and *OsARF25*, control the tiller angle (Li et al. 2020). However, the full roles of miR167 in the regulation of agronomic traits are largely unclear.

The phytohormone auxin is known to play an essential role in regulating agricultural traits in rice, including root system, tillers, panicle architecture, flower development, and seed development (Wang et al. 2017). For example, the overexpression of *OsAUX1*, the putative auxin influx carrier gene, led to an increase in the number of lateral roots (Zhao et al. 2015). Tillering in rice is affected by an alteration in auxin signaling. More tillers were produced following the accumulation of miR393, which targets the *OsTIR1*/*OsAFB* auxin receptor genes (Xia et al. 2012). Loss of function in the auxin biosynthesis gene *FIB* led to small panicles and a reduced number of spikelets (Yoshikawa et al. 2014).

ARFs belong to a transcription factor family that play crucial roles in auxin-signaling pathway through their specifical binding to auxin response elements (AuxRE) in the promoter of auxin response genes (Li et al. 2016). Based on genome-wide analysis, *ARF*s from 15 plant species have been identified (Li et al. 2016). For example, 22 *ARF* genes and a pseudogene have been identified in Arabidopsis, and 25 genes have been identified in rice (Li et al. 2016). It has been reported that *ARF*s play an essential role in several aspects of plant growth and development. In Arabidopsis, *ARF1* and *ARF2* are involved in controlling leaf senescence and the abscission of floral organs (Ellis et al. 2005). *ARF8* negatively regulates fertilization and fruit initiation (Goetz et al. 2006). In rice, *OsARF6* and *OsARF17* are involved in controlling flag leaf angle by regulating secondary cell wall biosynthesis of the lamina joints (Huang et al. 2021). *OsARF19* regulates rice leaf angles by positively regulating *Brassinosteroid Insenstive1* (*OsBRI1*) and *OsGH3-5* (Zhang et al. 2015a). In tomato (*Solanum lycopersicum* L.), *SlARF3* is reported to play multiple roles in development and is associated with the formation of trichomes and epidermal cells (Zhang et al. 2015b).

A rice spikelet contains a flower subtended by a pair of glumes that are aborted florets. Spikelets are the primary reproductive organs that determine grain yield. A rice flower consists of a lemma and palea, two lodicules, six stamens, and one pistil with two stigmas. Several genes involved in spikelet development have been characterized. For example, *OsMADS34*, a *SEPALLATA*-LIKE gene, controls rice inflorescence and spikelet development (Gao et al. 2010). The *SEPALLATA MADS* box gene *PAP2*, together with three *APETALA1* (*AP1*)-like genes, plays a role in the specification of inflorescence meristems (Kobayashi et al. 2012). *OsMADS1* functions in the development of rice flowers (Jeon et al. 2000). In addition, mutants showing cleistogamy have been characterized. A mutant lacking lodicules inside the spikelets showed cleistogamy (Maeng et al. 2006). A rice mutant *cl7*(t) developed by ethyl methanesulfonate (EMS) mutagenesis exhibited cleistogamy, contained thicker stems and more vascular bundles, had closed spikelets and a reduction in plant height (Ni et al. 2014). However, our knowledge of cleistogamy is limited.

In a previous study, we demonstrated that miR167d functions in rice immunity against *Magnaporthe oryzae* (Zhao et al. 2020). Here, we investigated the roles of miR167d in regulating flower opening and stigma size. First, we examined the flower opening and morphology of individual flower organs in overexpressing and blocking miR167d transgenic lines, including stigma size, elongation of stamen filaments, and morphological features of lodicules. Further, we obtained single or double mutants for the four *ARF*s that are the target genes of miR167d using CRISPR/Cas9 system or genetic cross. Then, we investigated the flower opening and morphology of individual flower organs in these mutants. Our data demonstrate that miR167d, together with four *ARF*s, had crucial roles in regulating stigma size, elongation of stamen filaments, and morphological features of lodicules. These findings establish a novel function of the miR167d-*ARF*s module in regulating flower opening and stigma size.

## Materials and Methods

### Plant Materials and Growth Conditions

The rice lines used in this study included OX167d, MIM167d (transgenic line expressing a target mimic of miR167d), *arf6*, *arf12*, *arf17*, *arf25*, *arf6 arf12*, *arf12 arf17*, and *arf12 arf25*. All these lines were in the Kasalath (KA, an indica cultivar) background. Besides, we also included OX167d in Zhonghua 11 backgrounds (ZH11, a japonica cultivar), which was generated by using the same construct and method as previously described (Zhao et al. 2020). OX167d/KA, MIM167d, *arf12*, and *arf25* were from a previous study (Zhao et al. 2020). The *arf6*, *arf17*, and *arf6 arf12* mutants were generated using CRISPR/Cas9 technology in the KA background. The *arf12 arf17* and *arf12 arf25* double mutants were obtained by genetic cross of *arf12-1* with *arf17-1* and *arf25-1* single mutant, respectively.

### CRISPR/Cas9 Plasmids Construction and Mutant Screening

To generate the *ARF6*, *ARF17*, and *ARF6 ARF12* knockout mutants, we constructed the plasmid as previously described (Xie et al. 2015) with slight modifications. Briefly, the target sites for each gene were screened and designed using the *CRISPR-PLANT* system (https://www.genome.arizona.edu/crispr/CRISPRsearch.html). The primers that contained the gene-specific sequences were designed using standards that were previously described (Xie et al. 2015). The DNA fragments to generate the *ARF6* knockout construction were amplified using the following primer pairs: PRGEB32-S5AD5-F/ARF6-gR1-R, ARF6-gR1-F/ARF6-gR2-R, and ARF6-gR2-F/PRGEB32-S3AD5-R. Together with the pRGEB32 plasmid, they were assembled following the manufacturer’s instructions for the NEB® Golden Gate Assembly Kit (New England Biolabs, Ipswich, MA, USA). During the assembly process, the pRGEB32 plasmid was used instead of pGGA plasmid in the kit. To generate the *ARF17* single and *ARF6 ARF12* double knockout constructs, we used the same methods as those for *ARF6* by just changing the gene-specific primers. The DNA fragments to generate the *ARF17* knockout construction were amplified using the following primer pairs: PRGEB32-S5AD5-F/ARF17-gR1-R, ARF17-gR1-F/ARF17-gR2-R, and ARF17-gR2-F/PRGEB32-S3AD5-R; The DNA fragments to generate the *ARF6 ARF12* double knockout construction were amplified using the following primer pairs: PRGEB32-S5AD5-F/ARF6/12-gR1-R, ARF6/12-gR1-F/ARF6/12-gR2-R, and ARF6/12-gR2-F/PRGEB32-S3AD5-R. All the positive constructs were confirmed using PCR and sequencing and were transformed into KA using *Agrobacterium*-mediated transformation with strain GV3101. To confirm the genotypes of knockout lines, all the transgenic T0 lines were verified using hygromycin (Hyg). DNA was then extracted from the Hyg-positive lines using the CTAB method and used for PCR amplification with gene-specific primers. The PCR products were sequenced, and the genotype of each mutation line was confirmed. All the primers for constructions are shown in Additional file 1: Table S1.

### Morphological Analysis and Microscopic Observations

The phenotypic characteristics of panicles, spikelets, and the floral organs were investigated using a DSLR camera (Nikon, Tokyo, Japan), an Olympus (Tokyo, Japan) stereoscope, and a microscope (Carl Zeiss AG, Jena, Germany), respectively. During the mature stage, the phenotypes of all the lines were investigated using a DSLR camera. The traits were measured as previously described (Li et al. 2022).

For paraffin sectioning, the spikelets from the different lines at the heading stage were fixed in FAA (50% [v/v] ethanol, 5% [v/v] acetic acid, and 3.7% [v/v] formaldehyde), vacuumed for 15 min, and incubated overnight at 4 ℃. The fixed spikelets were dehydrated by gradient ethanol, infiltrated with xylene, embedded into paraffin (Sigma-Aldrich, St. Louis, MO, USA), cut into thick slices, and dyed with 1% (w/v) safranin after they were pasted on microscope slides. Next, the slices were dewaxed, rehydrated, and dyed with 0.5% (w/v) toluidine blue. The microscopic observations were performed using a Zeiss microscope.

### RNA Isolation and Reverse Transcription-Quantitative Real-Time PCR (RT-qPCR) Assay

To detect the expression of miR167d and *ARF*s in OX167d/ZH11, we collected the plant leaf tissues at the seedling stage. Total RNA was extracted as previously described (Zhao et al. 2021; Hassan et al. 2022). cDNA was synthesized using NovoScript® Plus All-in-one 1st Strand cDNA Synthesis SuperMix (gDNA Purge) (Novoprotein, Shanghai, China). RT-qPCR was performed using Taq Pro Universal SYBR qPCR Master Mix (Vazyme, Nanjing, China) and the indicated primers to determine the abundance of mRNA on a Bio-Rad CFX96™ Real-Time system (Hercules, CA, USA). The abundance of gene expression was normalized using the ubiquitin (*UBI*) gene as an internal standard. The same method was used to determine the abundance of miR167d in ZH11 as previously described (Zhao et al. 2020). All the primers for the RT-qPCR assay are shown in Additional file 1: Table S1.

## Results

### Overexpression of miR167d Results in Cleistogamy

The miR167 family is conserved in planta based on annotations of the miRBase (https://www.mirbase.org/) (Kozomara et al. 2018), which is a searchable database of miRNA. There are 10 *MIR167* genes in rice that generate two types of mature miR167 sequences with only a single nucleotide difference at the 3’ end, i.e., miR167a-c and miR167d-j. In a previous study, we reported that miR167d functions in rice immunity against rice blast (Zhao et al. 2020). In this study, we describe our observation of the transgenic plants overexpressing miR167d (OX167d).

**Fig. 1:**
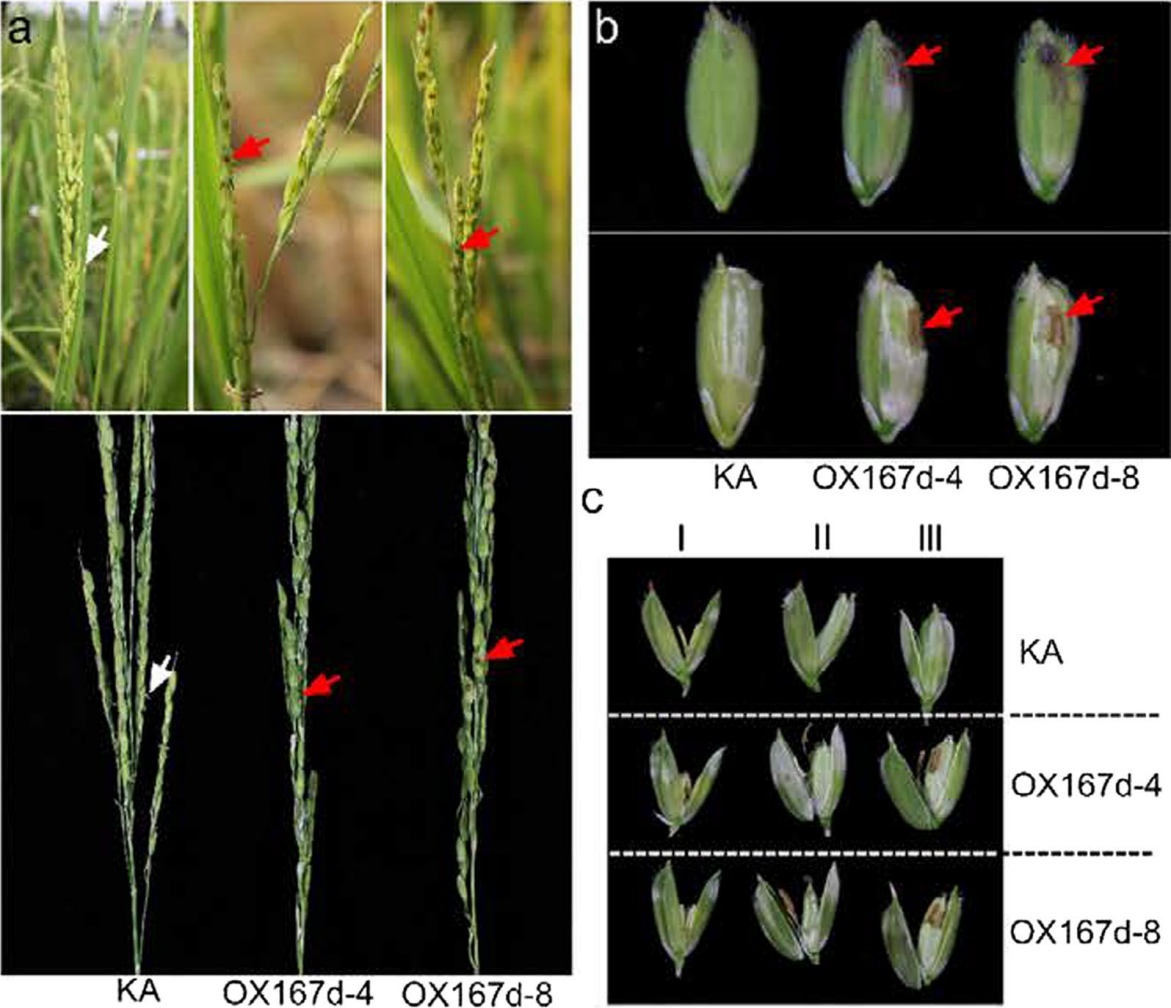
Phenotypic analysis of the spikelets and flowers in OX167d. **a** The inheritance of cleistogamy in the OX167d lines. The white arrows indicate the anthers that move out from the flowers. Red arrows indicate the stamens that remain inside the spikelets, forming the black dots. **b**, **c** The inner view of the flowers at the different stages. The early filling stage (**c** I), middle filling stage (**c** II), and late filling stage (**b**, **c** III). Red arrows indicate the stamens

One of the most striking phenotypes of the OX167d plants was cleistogamy. During flowering time, the anthers were not observed outside of the lemma and palea in OX167d compared with those in KA. Continual observation of spikelets from the heading to the filling stages indicated that the anthers were pushed out of the lemma and palea in KA flowers by the elongation of filaments and remained outside of them for a few days (Fig. 1a). In contrast, no anthers were observed outside of the lemma and palea in OX167d (Fig. 1a) except for the black spots inside the spikelets (Fig. 1a). When we peeled off parts of the lemma and palea in OX167d, the dead anthers were clearly displayed (Fig. 1b). The anthers always remained inside the spikelets even during the early, middle, and late filling stages of OX167d (Fig. 1c). Thus, these results suggest that the overexpression of miR167d results in cleistogamy.

In addition, the seedling growth and plant height were impacted by the overexpression of miR167d. The seedling height of the OX167d lines was significantly reduced compared with that of KA (Additional file 2: Fig. S1a). At the heading stage, the plant height of OX167d lines was reduced to approximately 50% that of KA (Additional file 2: Fig. S1b, c). Each of the four internodes was obviously shorter in OX167d than those in KA (Additional file 2: Fig. S1d). The OX167d lines showed larger tiller angles than those of KA at both the seedling and heading stages (Additional file 2: Fig. S1a, b, e, g), which were associated with the curve at the base of each tiller (Additional file 2: Fig. S1f). The number of tillers was significantly reduced in OX167d compared with KA (Additional file 2: Fig. S1h). Thus, our results suggest that overexpression of miR167d results in a reduction in plant height, larger tiller angle, and fewer tillers in rice.

Furthermore, the OX167d displayed aborted apical spikelets, panicle enclosure, and delayed maturity (Additional file 2: Fig. S2a). Moreover, the size of panicles was significantly reduced in OX167d compared with those in KA (Additional file 2: Fig. S2b, c). The seed setting rate was significantly reduced in OX167d compared with KA (Additional file 2: Fig. S2d). The 1,000-grain weight was decreased significantly in OX167d compared with that in KA (Additional file 2: Fig. S2e). The grain width and length in OX167d were comparable with those in KA (Additional file 2: Fig. S2f, h, j, l). However, the width and length of brown rice grain were significantly reduced compared with those in KA (Additional file 2: Fig. S2g, i, k, m). These results suggest that the overexpression of miR167d affects plant morphology and yield components.

### Overexpression of miR167d Alters the Elongation of Stamen Filaments, Stigma Size, and Lodicule

**Fig. 2:**
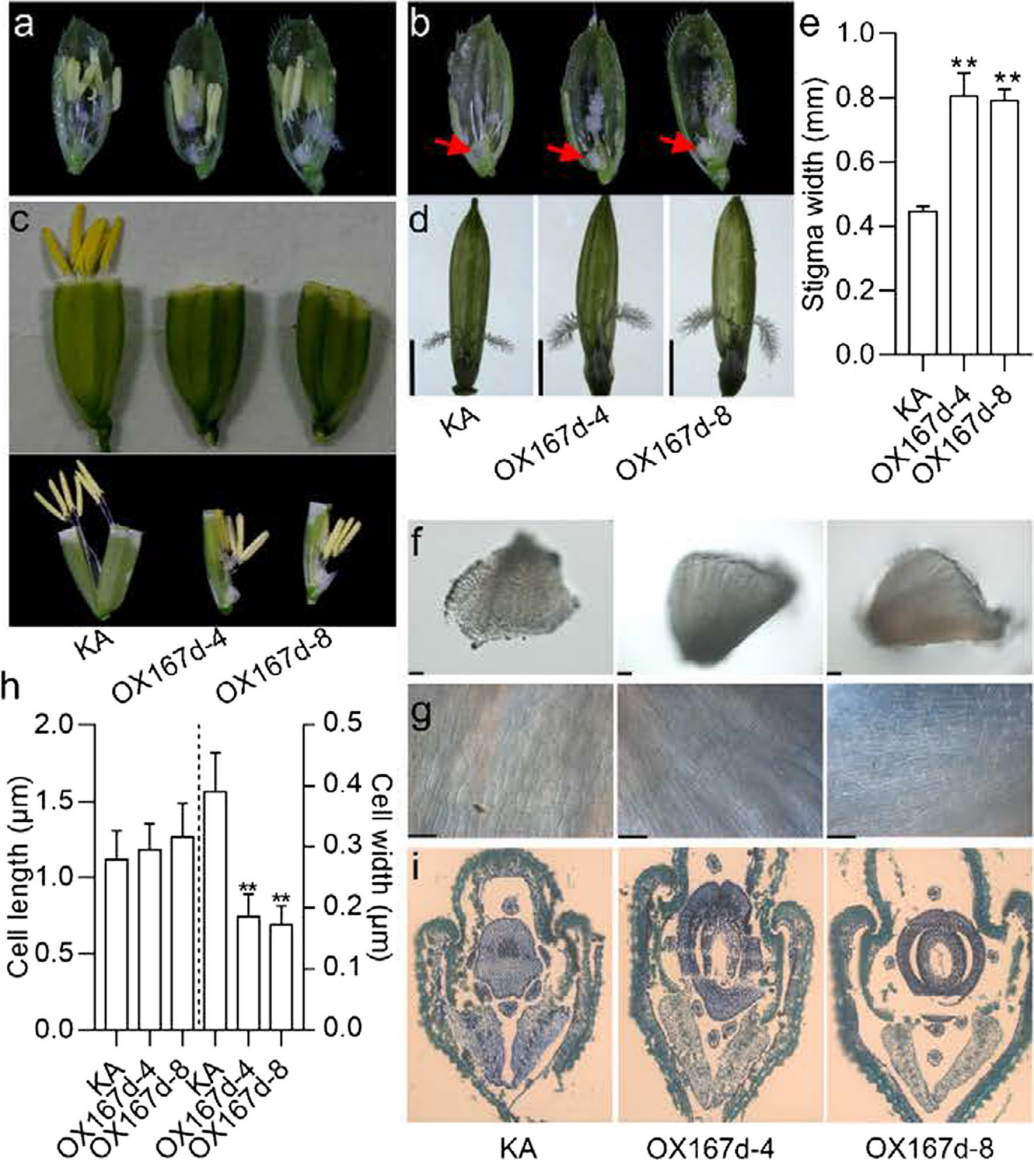
Phenotypic analysis of the floral organs in OX167d. **a** Overview of the inner floral organs in KA and OX167d lines. **b** Overview of the stigmas and lodicules. The stamen was removed from (**a**). Red arrows indicate lodicules. **c** The phenotype of the filament elongation in the indicated lines. **d** Close-up view of the stigmas. Bars, 2 mm. **e** Comparison of the stigma width in the indicated lines. Error bars indicate the standard deviation (SD) (n = 10). **P < 0.01 (Student’s *t*-test). **f** Close-up view of the lodicules in KA and OX167d. Bars, 100 μm. **g** Cell morphological features of lodicules in KA and OX167d. Bars, 50 μm. **h** Comparison of the cell length and cell width from (**g**). Error bars indicate standard deviation (SD) (n = 20). **P < 0.01 (Student’s *t*-test). **i** Transverse section of the flowers in KA and OX167d

To elucidate the reason for the cleistogamy of OX167d flowers, we examined the floral organs in detail. The stamens of OX167d had no apparent significant difference from that of KA at the heading stage (Fig. 2a), except that the lodicules in OX167d seemed white in contrast to the watery and transparent lodicules in KA (indicated by the red arrows) (Fig. 2b). However, the stamen filament failed in elongation at the flowering stage in OX167d (Fig. 2c). The size of stigma significantly increased in OX167d compared with that in KA (Fig. 2d, e). Since the lodicule enables flowers to open by swelling to push the lemma and the palea (Honda et al. 2005), its morphology is associated with cleistogamy (Yoshida et al. 2007). The microscopic observations showed that the size of the lodicule in OX167d was comparable with that in KA (Fig. 2f). However, the cell arrangement of lodicules in OX167d was significantly crowded compared with that in KA (Fig. 2g), and the cell width significantly decreased in OX167d, but not the cell length (Fig. 2h). The cross-sections revealed that OX167d showed a narrow shape, but the KA showed the plump shape of lodicule (Fig. 2i). Therefore, the cleistogamy of OX167d resulted from the defective lodicule. Collectively, overexpression of miR167d leads to defects in stamen filament elongation and lodicules narrowing, but increased stigma size.

To exclude that the effects of miR167d on flower opening and stigma size are dependent on genetic background, we constructed the overexpressing miR167d lines in ZH11 (a japonica cultivar) (hereafter, named OX167d/ZH11) and obtained 17 transgenic lines that showed the same phenotypes. The plant height was significantly reduced compared with that of ZH11 (Additional file 2: Fig. S3a). The lengths of four internodes from the top were shorter in OX167d/ZH11 than in ZH11 (Additional file 2: Fig. S3b). Thus, we selected three lines in which there was a significant increase in the accumulation of mature miR167d for further study (Additional file 2: Fig. S3c). As previously described (Zhao et al. 2020), miR167d suppresses its target genes at the post-transcriptional level (Zhao et al. 2020). We examined the expression of miR167d target genes and found that the abundance of mRNA for each gene was significantly reduced compared with that in ZH11 (Additional file 2: Fig. S3d), indicating that miR167d was successfully overexpressed and functioned.

Next, we examined the inner floral organs in detail. The stamens in OX167d/ZH11 had no apparent difference from those of ZH11 (Additional file 2: Fig. S4a). However, the stigma size of OX167d/ZH11 significantly increased (Additional file 2: Fig. S4b), and the stamen filament failed in elongation compared with that in ZH11 at the flowering stage (Additional file 2: Fig. S4c, d). In addition, the anthers remained inside the spikelets (Additional file 2: Fig. S4e). These results indicate that the influence of miR167d on flower opening and stigma size is independent on genetic background. Therefore, we used KA to conduct in-depth research.

### Blocking miR167d by Target Mimicry Results in Morphological Alteration of Stigma and Lodicule

**Fig. 3:**
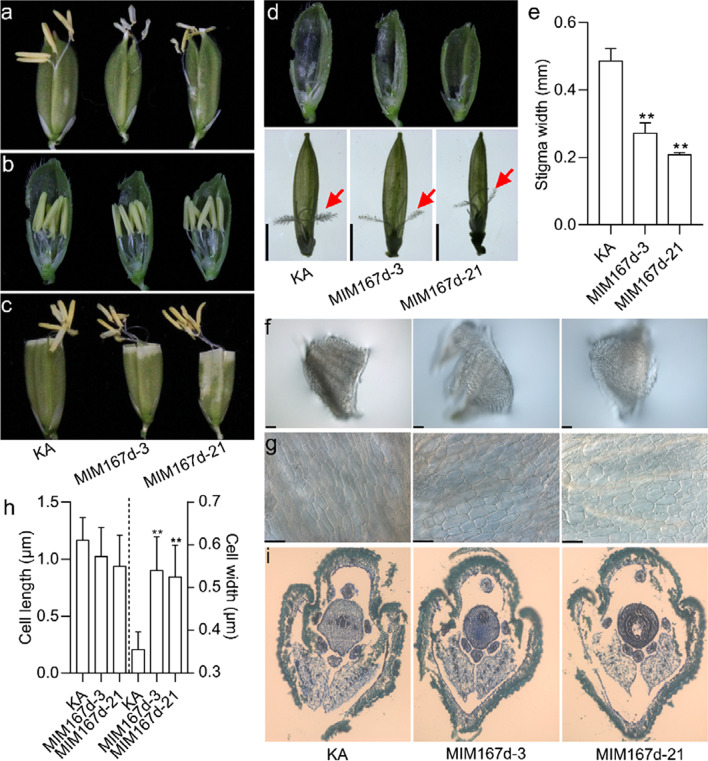
Phenotypic analysis of the floral organs in MIM167d. **a** Phenotype of the flowers. The anthers from KA and MIM167d move out from the flowers. **b** Overview of the inner floral organs in KA and MIM167d lines. **c** Phenotype of the filament elongation. **d** Phenotype of the stigmas. The stamen was removed from (**b**). Red arrows indicate the stigmas. Bars, 2 mm. **e** Comparison of the stigma width. Error bars indicate the standard deviation (SD) (n = 10). **P < 0.01 (Student’s *t*-test). **f** Close-up view of the lodicules in KA and MIM167d. Bars, 100 μm. **g** Cell morphological features of lodicules in KA and MIM167d. Bars, 50 μm. **h** Comparison of the cell length and cell width from (**g**). Error bars indicate the standard deviation (SD) (n = 20). **P < 0.01 (Student’s *t*-test). (**i**) Transverse sections of flower in KA and MIM167d

To further clarify the functions of miR167d in flower opening and stigma size, we used two independent transgenic lines designated MIM167d that overexpressed a target mimic of miR167d from a previous study (Zhao et al. 2020), which led to a significant reduction in the accumulation of miR167d. In MIM167d and KA flowers, the anthers were pushed out of the lemma and palea by the elongation of filaments (Fig. 3a), indicating normal flowering. The anthers of MIM167d exhibited no significant difference from those of KA (Fig. 3b). The filament elongation of MIM167d was comparable to that of KA (Fig. 3c). However, the size of stigma in MIM167d was significantly reduced compared with that in KA (Fig. 3d, e). In addition, microscopic observation showed that the size of the lodicule in MIM167d was comparable with that in KA (Fig. 3f). However, the cell width of the lodicule, but not the cell length, was significantly increased in MIM167d compared with that in KA (Fig. 3g, h). The cross-sections revealed that the shape of lodicule in MIM167d was similar to that in KA (Fig. 3i). These results suggest that blocking miR167d results in morphological alteration of stigma and lodicule but cannot result in cleistogamy in rice.

In addition, the survey of agronomic traits demonstrated that the plant height of MIM167d was significantly reduced compared with that in KA (Additional file 2: Fig. S5a, b). The tiller angles were comparable with those of KA (Additional file 2: Fig. S5c, d). However, the tiller number of MIM167d was significantly higher than that of KA (Additional file 2: Fig. S5e). The size of the panicles was significantly smaller in MIM167d than in KA (Additional file 2: Fig. S6a, b). The seed setting rate and 1,000-grain weight were significantly decreased in MIM167d compared with those in KA (Additional file 2: Fig. S6c, d), leading to straight panicles at the mature stage (Additional file 2: Fig. S5a). In addition, the width and length of grain and brown rice grain were significantly reduced in MIM167d compared with those in KA (Additional file 2: Fig. S6e-h, i-l). These results suggest that blocking miR167d causes defects in agronomic traits.

### Four *ARF* Genes have Overlapping Functions in Flower Opening and Stigma Size

**Fig. 4:**
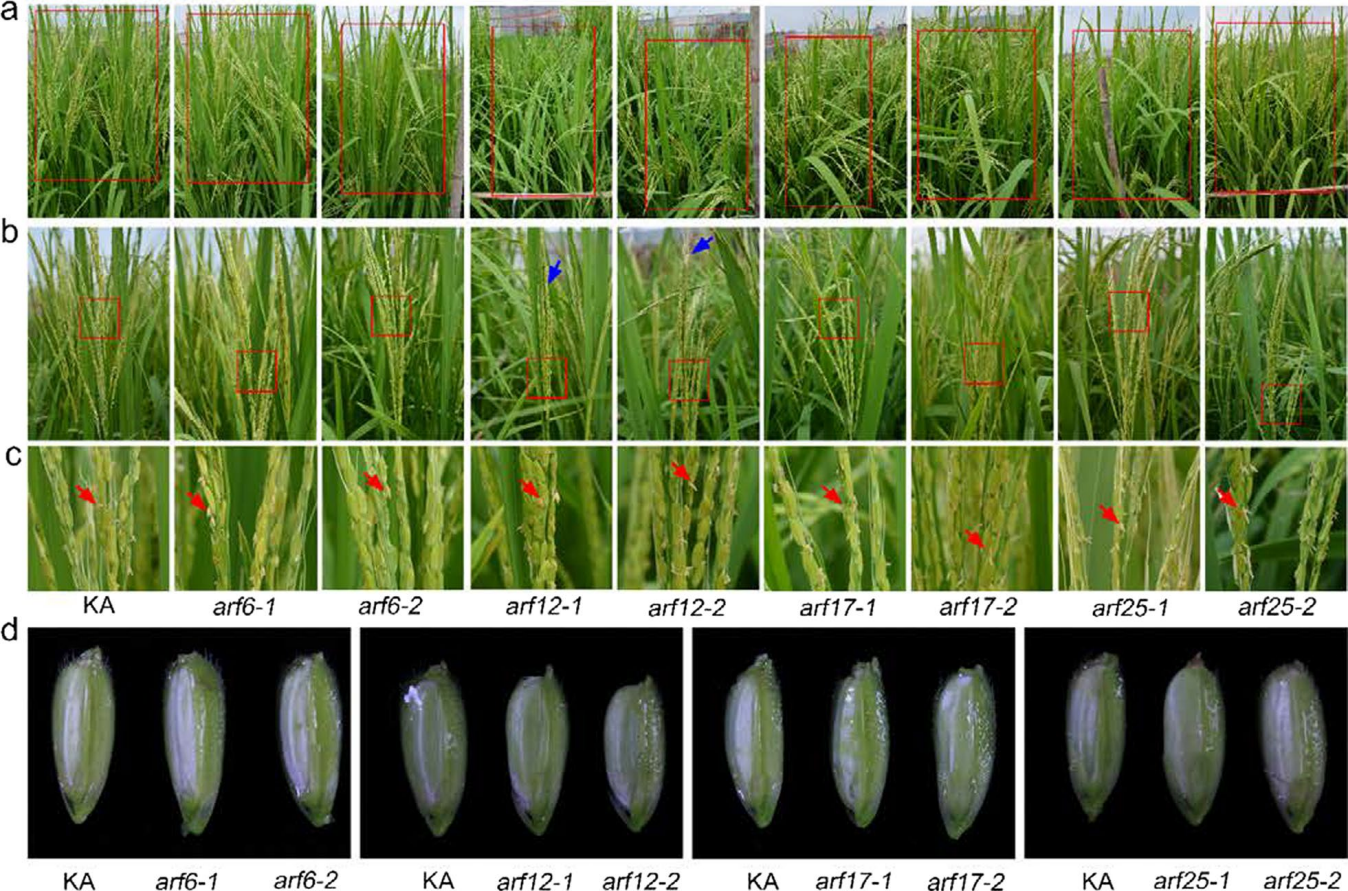
Panicle and flower phenotypes of the *ARF* single mutants. **a**-**c** Panicles of KA and *ARF* single mutants. Overview of the panicles (**a**), Close-up view of the panicles (**b**, **c**). Blue arrows indicate aborted apical spikelets. Red arrows indicate the anthers that move out from the flowers. **d** The inner view of the flowers at the later grain filling stage

**Fig. 5:**
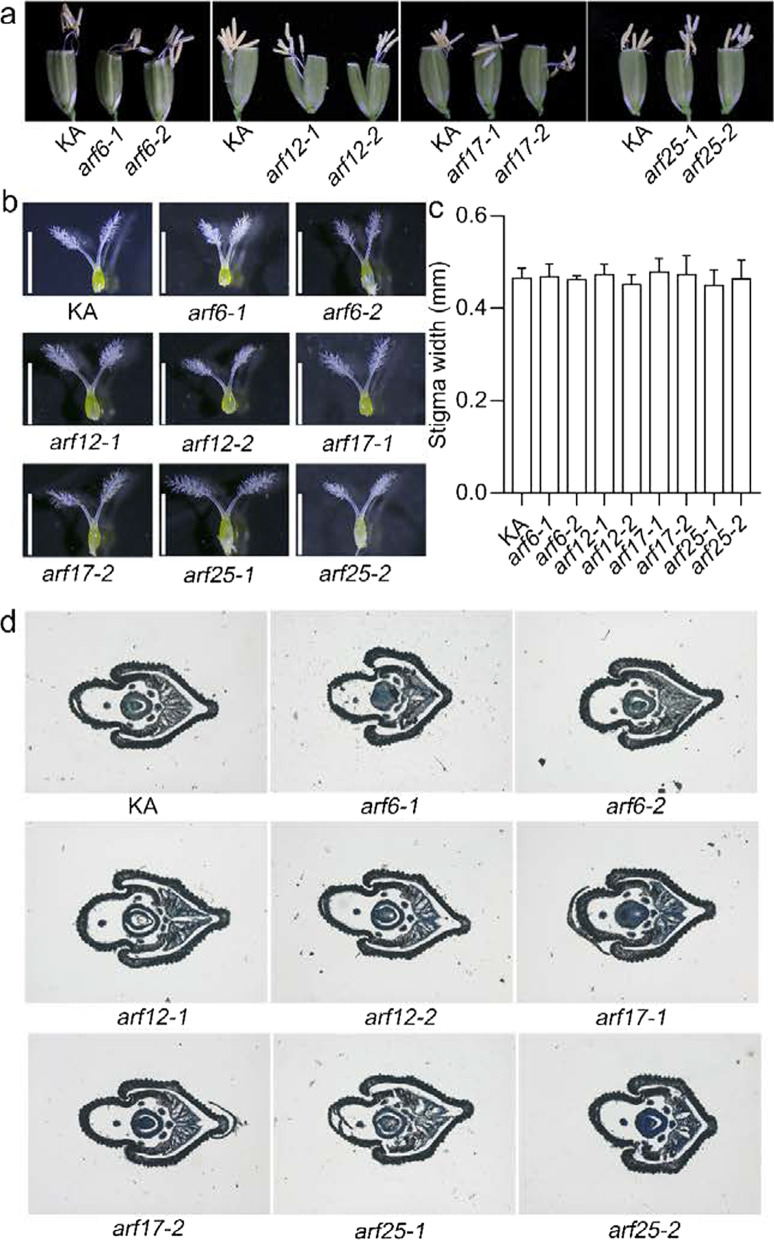
Phenotypic analysis of the floral organs in *ARF* single mutants. **a** The phenotype of the filament elongation in the indicated lines. **b** Phenotype of the pistils. Bars, 2 mm. **c** Comparison of the stigma width. Error bars indicate the standard deviation (SD) (n = 10). **(P < 0.01) (Student’s *t*-test). **d** Transverse sections of flower in KA and *ARF* single mutants

Previous studies have shown that miR167d has four target genes, namely *ARF6*, *ARF12*, *ARF17*, and *ARF25*, which encode auxin response factors (Zhao et al. 2020). This prompted us to identify which target genes function in flower opening and stigma size, particularly those that result in cleistogamy. Therefore, we generated single mutants for each of these four *ARF* genes. Among them, *arf12-1*, *arf12-2*, *arf25-1*, and *arf25-2* were created in a previous study (Zhao et al. 2020). The mutants of *ARF6* and *ARF17* were constructed using CRISPR/Cas9 technology (Additional file 2: Fig. S7). Fortunately, we obtained two independent homozygous mutants for each gene, including *arf6-1* and *arf6-2* for *ARF6*, and *arf17-1* and *arf17-2* for *ARF17* (Additional file 2: Fig. S7). All the mutants harbored deletions that resulted in a frameshift and caused protein truncation (Additional file 2: Fig. S7). Next, we assessed the phenotype of spikelet organs in these mutants. In KA and all the *ARF* single mutant flowers, the anthers were out of the lemma and palea and remained outside (Fig. 4a–c), and no anthers remained inside the spikelets (Fig. 4d). The elongation of filaments was normal in the single mutants compared with that in KA (Fig. 5a). Microscopic observation showed that the size of stigma in the *ARF* single mutants was comparable with that in KA (Fig. 5b, c). The cross-sections revealed that the shape of the lodicule in the *ARF* single mutants was similar to that in KA (Fig. 5d). Thus, these results suggest that *ARF6*, *ARF12*, *ARF17*, and *ARF25* may have functional redundancy in regulating flower opening and stigma size.

In addition, the plant height and the number of tillers per plant were similar in the *arf6*, *arf17*, and *arf25* mutants compared with those in KA (Additional file 2: Fig. S8). However, the plant height of the *arf12* mutants was significantly reduced compared with that of KA (Additional file 2: Fig. S8a, b), but there were no significant differences in the tiller numbers (Additional file 2: Fig. S8c). Meanwhile, the *arf12* mutants displayed aborted apical spikelets (Fig. 4b). These results indicate that *ARF12* may play a major role in plant height and the development of spikelets among the *ARF*s targeted by miR167d.

**Fig. 6:**
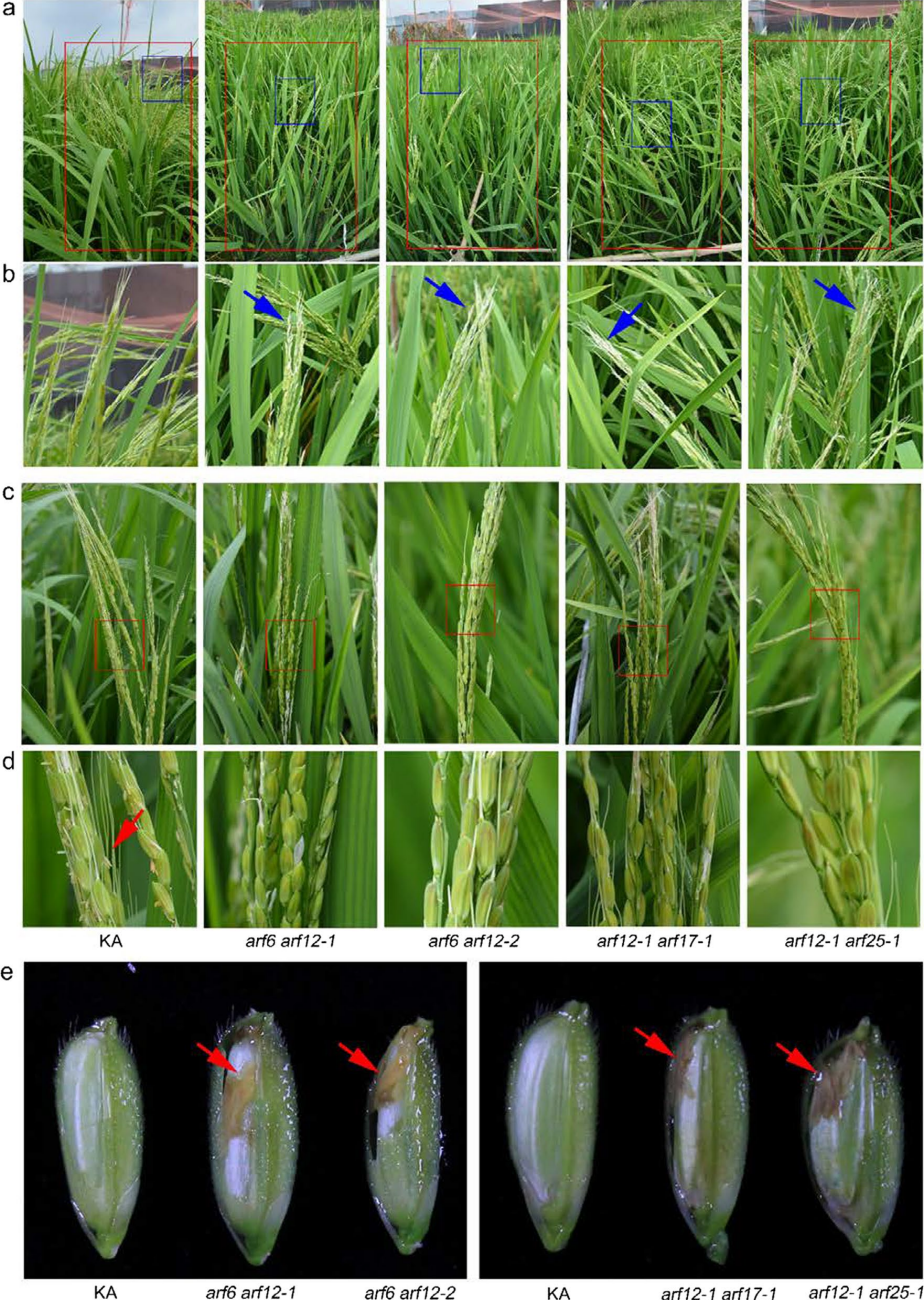
Panicle and flower phenotypes of the *ARF* double homozygous mutants. **a**-**c** Panicles of KA and *ARF* double mutants. Overview of the panicles (**a**). Close-up view of the panicles (**b**–**c**). Blue arrows indicate aborted apical spikelets. **d** Zoom-in view of the red box in (**c**). Red arrows indicate the anthers that move out from the flowers. **e** The inner view of the flowers at the later grain filling stage. Red arrows indicate stamens

**Fig. 7:**
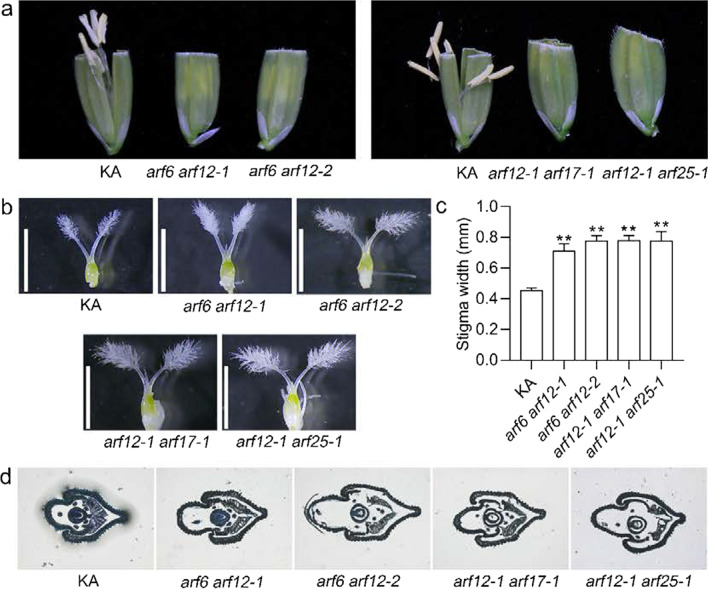
Phenotypic analysis of the floral organs in *ARF* double mutants. **a** The phenotype of the filament elongation in the indicated lines. **b** Phenotype of the pistils. Bars, 2 mm. **c** Comparison of the stigma width. Error bars indicate the standard deviation (SD) (n = 10). **P < 0.01 (Student’s *t*-test). **d** Transverse sections of flower in the KA and *ARF* double homozygous mutants

To confirm whether the four *ARF*s were functionally redundant, we constructed double mutants of the *ARF* genes, i.e., *arf6 arf12*, *arf12 arf17*, and *arf12 arf25*, because only the *ARF12* single mutant lines resulted in defects in development of spikelets and the plant height. Among the double mutants, *arf12-1 arf17-1* and *arf12-1 arf25-1* were obtained by genetic cross using *arf12-1*, *arf17-1*, and *arf25-1* single mutants. *arf6 arf12* was constructed using CRISPR/Cas9 technology. We obtained two independent double homozygous mutants, i.e., *arf6 arf12-1* and *arf6 arf12-2* (Additional file 2: Fig. S7). Both of the mutant lines harbored deletion or insertion that resulted in a frameshift and caused the proteins to be truncated (Additional file 2: Fig. S7). Consistently, the aborted apical spikelets were observed in all the double homozygous mutants compared with that in KA (Fig. 6a, b). Moreover, no anthers were observed outside of the lemma and palea (Fig. 6c, d) but remained inside the spikelets in the double mutant (Fig. 6e). Conversely, the stamen filaments elongated out of the lemma and palea in KA (Fig. 6c, d). In addition, the filaments of all three double mutants failed in elongation at the flowering stage (Fig. 7a). Microscopic observations showed that the size of the stigma had increased significantly compared with that of KA (Fig. 7b, c). The cross-sections revealed that the double mutants showed a narrow, but the KA showed a plump lodicule (Fig. 7d). These results suggest that *ARF6*, *ARF12*, *ARF17*, and *ARF25* have an overlapping function in regulating flower opening, stigma size, and cleistogamy.

Furthermore, we assessed the plant phenotypes of these double mutants. It showed that plant height and the number of tillers per plant were significantly reduced in the double mutants compared with those in KA (Additional file 2: Fig. S9). These results suggest that *ARF6*, *ARF12*, *ARF17*, and *ARF25* have an overlapping function in regulating plant height and tiller number in rice.

## Discussion

### The miR167d-*ARF*s Module is Crucial to Regulate Flower Opening and Stigma Size

miR167 is ubiquitous across all the terrestrial plant species from ferns to monocotyledons and eudicotyledons, and it is generally highly expressed next only to miR156 and miR166 (Chávez Montes et al. 2014). This suggests that miR167 may have conservative and crucial functions. Flower opening is essential for cross-pollination in seed production of hybrid rice. A previous study suggested that miR167 regulates pistil development by targeting *InARF8* in Japanese morning glory (*Ipomoea nil*) (Glazińska et al. 2014). In this study, we identified the miR167d-*ARF*s as key regulatory elements in flower opening and stigma size in rice. Overexpression of miR167d led to increased stigma size and cleistogamy that is associated with failure of filament elongation and morphological alteration of lodicule (Figs. 1 and 2). In contrast, blocking miR167d led to decreased stigma size and alteration of lodicule cell morphology (Fig. 3). In addition, double mutants of *ARF*s showed morphological alteration similar to those of OX167d compared with wild type (Figs. 6 and 7). Thus, the miR167d-*ARF*s module is crucial to regulating the flower opening and stigma size.

In flowering plants, anther dehiscence following the release of pollen is essential for sexual reproduction. In Arabidopsis, miR167 and its target genes, *ARF6* and *ARF8*, function in anther growth and dehiscence (Zheng et al. 2019). Thus, future studies should focus on examining the function of the miR167d-*ARF*s module in anther growth and dehiscence in rice.

### *ARF6*,* ARF12*, *ARF17*, and *ARF25* have Overlapping Functions

There are 25 *ARF*s genes in the rice genome (Wang et al. 2007). These genes encode the auxin response factors that specifically bind to the AuxREs found in the promoters of auxin response genes that contain TGTCTC. These ARFs contain a conserved DNA binding domain at the N-terminal, a central region, and a protein–protein interaction domain at the C-terminal (Wang et al. 2007; Li et al. 2020). The function of most remains unclear (Li et al. 2020). In this study, we showed that *ARF6*/*12*/*17*/*25* have overlapping functions in regulating flower opening, stigma size, and other agronomic traits in rice.

Single knockout mutants for these four *ARF*s showed the normal flower opening and filament elongation, and comparable stigma size with KA (Figs. 4 and 5). In addition, the agronomic trait survey showed that the plant height and tiller number in the mutants were similar to those in KA (Fig. S8), except for *arf12*, which showed a significant reduction in plant height and aborted apical spikelets (Fig. 4b and Additional file 2: Fig. S8), indicating that *ARF12* has a major role in regulating plant height and apical spikelet development among these *ARF*s. However, a previous study showed that the plant height of *arf12* in the ZH11 background did not differ significantly compared with that of the WT (Li et al. 2020). We hypothesized that this could result from the difference in plant heights between KA and ZH11. Thus, the *arf12* mutant in ZH11 did not show a significant difference in plant height compared with that of ZH11.

These four *ARF*s belong to the same subgroup in rice (Wang et al. 2007), also suggesting they are redundantly functional. In this study, mutation in *ARF12* together with the mutation in either *ARF6*, *ARF17*, or *ARF25* exhibited aborted apical spikelets, failed elongation of stamen filaments, increased stigma size, morphological alteration of lodicule, and cleistogamy (Figs. 6 and 7). In addition, the plant height and tillers per plant were significantly reduced in the double homozygous mutants compared with those in KA (Additional file 2: Fig. S9). Therefore, *ARF6*, *ARF12*, *ARF17*, and *ARF25* have overlapping functions in flower opening, stigma size, plant height, and tiller number in rice.

### Cleistogamy may be due to the Modulation of the Lodicule Development Genes

Cleistogamy is defined as fertilization without flower opening (Ohmori et al. 2018). It is an effective strategy to prevent “gene flow” from the cultivated species to their wild relatives and helps the plants to survive under unfavorable conditions (Yoshida et al. 2007; Lombardo et al. 2017). It has been reported that altering lodicule morphology can result in cleistogamy because the defective organs are unable to exert sufficient outward pressure to trigger the flower opening (Lombardo et al. 2017). In this study, we demonstrated that overexpressing miR167d or knocking out *ARF6 ARF12*, *ARF12 ARF17*, and *ARF12 ARF25* results in morphological alteration of the lodicule and leads to cleistogamy (Figs. 1, 2, 6 and 7). To our knowledge, this study represents the first characterization of a miR167d-*ARF*s module involved in cleistogamy in rice.

To date, more than 10 genes have been identified that are involved in cleistogamy. However, only two of them had been cloned, including *SUPERWOMAN 1* (*SPW1*) (Yoshida et al. 2007) and *CL7(t)*/*DEP2* (Ni et al. 2014). *SPW1* is a class-B MADS-box gene, which specifies the identities of lodicules and stamens (Yoshida et al. 2007), and *CL7(t)* could participate in the development of lodicules (Ni et al. 2014). Based on genetic and molecular studies, several MADS-box transcription factors were involved in the development of lodicules, including *OsMADS1*, *OsMADS2*, and *OsMADS3* (Yoshida et al. 2007). Suppression of *OsMADS2* expression results in flowers that do not open (Yadav et al. 2007), suggesting that manipulating the expression of lodicule development genes could result in cleistogamy. Thus, future studies should focus on examining the function of *ARF*s in regulating lodicule development genes.

### The Appropriate Expression of miR167d is Crucial for Agronomic Traits

It has been reported that miR167 is involved in the regulation of plant vegetation, flowering time, reproductive organ development, and stress response by the regulation of its target genes, which encode the ARFs (Liu et al. 2021). For example, in Arabidopsis, miR167 targets *ARF6* and *ARF8* and controls somatic embryogenesis, seed development, and adventitious rooting (Gutierrez et al. 2009; Su et al. 2016; Yao et al. 2019). Overexpression of target mimics of miR167 results in a late flowering phenotype (Todesco et al. 2010). In rice, overexpression of miR167b results in a decreased abundance of mRNA of the four *ARF* genes, and the transgenic lines were small in stature and had remarkably reduced tiller numbers (Liu et al. 2012). In tomatoes, the downregulation of *ARF6* and *ARF8* by miR167 leads to female sterility and defects in floral development (Liu et al. 2014). In Japanese morning glory (*Ipomoea nil*), miR167 and *InARF8* participate in vegetative and generative development (Glazińska et al. 2014). In this study, we identified miR167d as a key regulator of agronomic traits in rice.

Previous studies suggest that the overexpression of miR167a in the ZH11 background results in reduced plant height, fewer tiller numbers, and larger tiller angles (Li et al. 2020). Overexpression of miR167b in the cultivar Nipponbare resulted in a shorter stature and fewer tillers (Liu et al. 2012). In this study, similar phenotypes were observed in the miR167d lines that were overexpressed in the KA background (Additional file 2: Fig. S1). In addition, we observed that the overexpression or inhibition of the abundance of miR167d affected rice agronomic traits, including plant height, the tiller number, panicle and spikelet development, grain width and length, seed setting rate, and 1,000-grain weight (Additional file 2: Figs. S1, S2, S5 and S6). Among them, some agronomic traits were defective in both OX167d and MIM167d. For example, the plant height was significantly reduced in both OX167d and MIM167d (Additional file 2: Figs. S1 and S5). Yield-related traits were defective in both lines (Additional file 2: Figs. S2 and S6). Thus, our research demonstrates that the appropriate expression of miR167d is crucial for agronomic traits.

### miR167d may Function Differently from miR167a-c and/or in Different Genetic Backgrounds

Though miR167 is a highly conserved miRNA family in plants, the number of *MIR167* genes are various among different plant species. For example, there are four *MIR167* genes in Arabidopsis, designated *MIR167a* to *MIR167d*, and miR167a is highly abundant and acts as a key regulator in the development of female and male organs (Yao et al. 2019). There are 10 *MIR167* genes in rice, each of which has different abundance. *MIR167a-c* probably plays a primary role because the abundance of miR167a-c was higher than that of miR167d-j when the genomic sequences covering the stem-loop region of each *MIR167* gene were transiently expressed in tobacco leaves (Liu et al. 2012). However, miR167d-j is induced higher than miR167a-c in Lijiangxin Tuan Heigu upon biotic stress (Li et al. 2014). Previous studies suggested that miR167a overexpression lines produced longer grains and had increased 1,000-grain weight in the WT/DJ background (Qiao et al. 2021). The overexpression of *MIM167a* has no effect on the plant height in the ZH11 background (Li et al. 2020). However, in this study, overexpressing miR167d lines significantly reduced the 1,000-grain weight in the KA background (Additional file 2: Fig. S2). Moreover, the brown rice grain width and length were significantly reduced compared with those of KA (Additional file 2: Fig. S2), and the MIM167d lines significantly reduced the plant height (Additional file 2: Fig. S5a, b). Thus, miR167d may function differently with miR167a-c and/or in different genetic backgrounds.

## Conclusions

In the present study, we demonstrated that overexpressing miR167d, or knockout of *ARF12*, together with *ARF6*, *ARF17*, or *ARF25*, resulted in failed elongation of stamen filaments, increased stigma size, morphological alteration of lodicule. In contrast, blocking miR167d expression led to a reduction in stigma size and alteration of lodicule cell morphology. Furthermore, *ARF6*, *ARF12*, *ARF17*, and *ARF25* have overlapping functions in flower opening and stigma size. Thus, appropriate expression of miR167d and miR167d-*ARF*s module are crucial for agronomic traits in rice.

## Supplementary Information

**Additional file 1**: **Table S1**. The Primers used in this study.

**Additional file 2**: **Fig. S1**. Phenotypes of OX167d in the KA background. a, b Plant morphology of KA and OX167d lines at the seedling stage (a) and heading stage (b). c Comparison of the plant height between KA and OX167d lines. d Comparison of the internode length. e Vertical observation. f The curve observation. g, h Comparison of the tillers angle (g) and tiller number (h). In (c), (g), (h), error bars indicate the standard deviation (SD) (n=10). **P<0.01 (Student’s t-test). **Fig. S2**. Panicle morphology and yield components of OX167d in the KA background. a, b Panicle morphology of the indicated lines. Pictures were taken before (a) and at the mature stage (b). In (a), red arrows indicate panicle enclosure, and white arrows indicate aborted apical spikelets. Bar, 8 cm in (b). c-e Comparison of the panicle length (c), seed setting rate (d), and 1,000-grain weight (e). f-i Analysis of grain and brown rice grain phenotypes in the indicated lines. Grain width (f), brown rice grain width (g), grain length (h), and brown rice grain length (i). j-m Comparison of the grain width (j), brown rice grain width (k), grain length (l), and brown rice grain length (m) in the indicated lines. In (c-e, j-m), error bars indicate the standard deviation (SD) (n=10). **P<0.01 (Student’s t-test). **Fig. S3**. Phenotypes of OX167d in the ZH11 background. a Plant morphology of ZH11 and OX167d lines at the heading stage. b The internode length comparison of ZH11 and OX167d lines. c Analysis of the expression of miR167d in the indicated lines by RT-qPCR. U6 was used as an internal reference. d Relative mRNA abundance of the genes targeted by miR167d in the indicated lines. ARF6: Os02G0164900; ARF12: Os04g0671900; ARF17: Os06g0677800; ARF25: Os12g0613700. In (c, d), error bars indicate the standard deviation (SD) (n=3). **P<0.01 (Student’s t-test). **Fig. S4**. Spikelet characteristics of ZH11 and OX167d lines. a, b Spikelets of the ZH11 and OX167d lines. The stamen was removed in (b) from (a). Red arrows indicate stigmas. c, d Comparison of filament elongation in the indicated lines. e Comparison of the inner organs at the grain filling stage. Parts of the palea and lemma have been removed to allow the observation of inner organs. White arrows indicate the stigmas, and green arrows indicate the stamens. **Fig. S5**. Phenotypes of MIM167d in the KA background. a Plant morphology of KA and MIM167d lines at the heading stage. b Comparison of the plant height. c The curve observation. d, e Comparison of the tillers angle (d) and tiller number (e) in the indicated lines. In (b, d, e), error bars indicate the standard deviation (SD) (n=10). **P<0.01 (Student’s t-test). **Fig. S6**. Panicle morphology and yield components in the MIM167d lines. a Panicle morphology of KA and MIM167d lines. Pictures were taken at the mature stage. Bar, 8 cm. b-d Comparison of the panicle length (b), seed setting rate (c), and 1,000-grain weight (d) of the indicated lines. e-h Analysis of grain and brown rice grain phenotypes in the indicated lines. Grain width (e), brown rice grain width (g), grain length (f), and brown rice grain length (h). i-l Comparison of the grain width (i), grain length (j), brown rice grain length (k), and brown rice grain width (l) in the indicated lines. In (b-d, i-l), the error bars indicate the standard deviation (SD) (n=10). **P<0.01, *P<0.05 (Student’s t-test). **Fig. S7**. The genotypes of the indicated knockout mutants. Alignment of the gDNA sequences around the gRNA sites in the indicated mutants. Two typical lines were obtained for each single or double mutant. The target sites are highlighted in yellow. The protospacer adjacent motifs are highlighted in red. Numbers on the right side indicate the deleted or inserted base pairs of the mutant lines indicated. **Fig. S8**. Phenotypes of ARF single mutants. a Plant morphology of the indicated lines at the heading stage, including arf6, arf12, arf17, and arf25. b, c Comparison of the plant height (b) and tiller number (c) in the indicated lines. Error bars indicate the standard deviation (SD) (n=10). **P<0.01 (Student’s t-test). **Fig. S9**. Phenotypes of ARF double mutants. a Plant morphology of the indicated lines at the heading stage, including arf6 arf12, arf12 arf17, and arf12 arf25. b, c Comparison of the plant height (b) and tiller number (c) in the indicated lines. Error bars indicate the standard deviation (SD) (n=10). **P<0.01 (Student’s t-test).

## Data Availability

Not applicable.

## Abbreviations

miRNA: MicroRNA
SPL: SQUAMOSA promoter-binding protein-like transcription factor
GRF: Growth-regulating factor
ARF: Auxin response factor
AuxRE: Auxin response elements
*AP1*: *APETALA1*
EMS: Ethyl methanesulfonate
KA: Kasalath
ZH11: Zhonghua 11
Hyg: Hygromycin
RT-qPCR: Reverse transcription-quantitative real-time PCR
