# Supplementary material for: miR167d-ARFs Module Regulates Flower Opening and Stigma Size in Rice
Source: Rice (N Y). 2022 Jul 25;15:40. doi: 10.1186/s12284-022-00587-z (PMC9314575; doi:10.1186/s12284-022-00587-z)
Supplement: Supplementary file 2 — Additional file 2: Fig. S1. Phenotypes of OX167d in the KA background. a, b Plant morphology of KA and OX167d lines at the seedling stage (a) and heading stage (b). c Comparison of the plant height between KA and OX167d lines. d Comparison of the internode length. e Vertical observation. f The curve observation. g, h Comparison of the tillers angle (g) and tiller number (h). In (c), (g), (h), error bars indicate the standard deviation (SD) (n=10). **P<0.01 (Student’s t-test). Fig. S2. Panicle morphology and yield components of OX167d in the KA background. a, b Panicle morphology of the indicated lines. Pictures were taken before (a) and at the mature stage (b). In (a), red arrows indicate panicle enclosure, and white arrows indicate aborted apical spikelets. Bar, 8 cm in (b). c-e Comparison of the panicle length (c), seed setting rate (d), and 1,000-grain weight (e). f-i Analysis of grain and brown rice grain phenotypes in the indicated lines. Grain width (f), brown rice grain width (g), grain length (h), and brown rice grain length (i). j-m Comparison of the grain width (j), brown rice grain width (k), grain length (l), and brown rice grain length (m) in the indicated lines. In (c-e, j-m), error bars indicate the standard deviation (SD) (n=10). **P<0.01 (Student’s t-test). Fig. S3. Phenotypes of OX167d in the ZH11 background. a Plant morphology of ZH11 and OX167d lines at the heading stage. b The internode length comparison of ZH11 and OX167d lines. c Analysis of the expression of miR167d in the indicated lines by RT-qPCR. U6 was used as an internal reference. d Relative mRNA abundance of the genes targeted by miR167d in the indicated lines. ARF6: Os02G0164900; ARF12: Os04g0671900; ARF17: Os06g0677800; ARF25: Os12g0613700. In (c, d), error bars indicate the standard deviation (SD) (n=3). **P<0.01 (Student’s t-test). Fig. S4. Spikelet characteristics of ZH11 and OX167d lines. a, b Spikelets of the ZH11 and OX167d lines. The stamen was removed in (b) from (a). Red ar [file 12284_2022_587_MOESM2_ESM.pptx]

## Slide 1
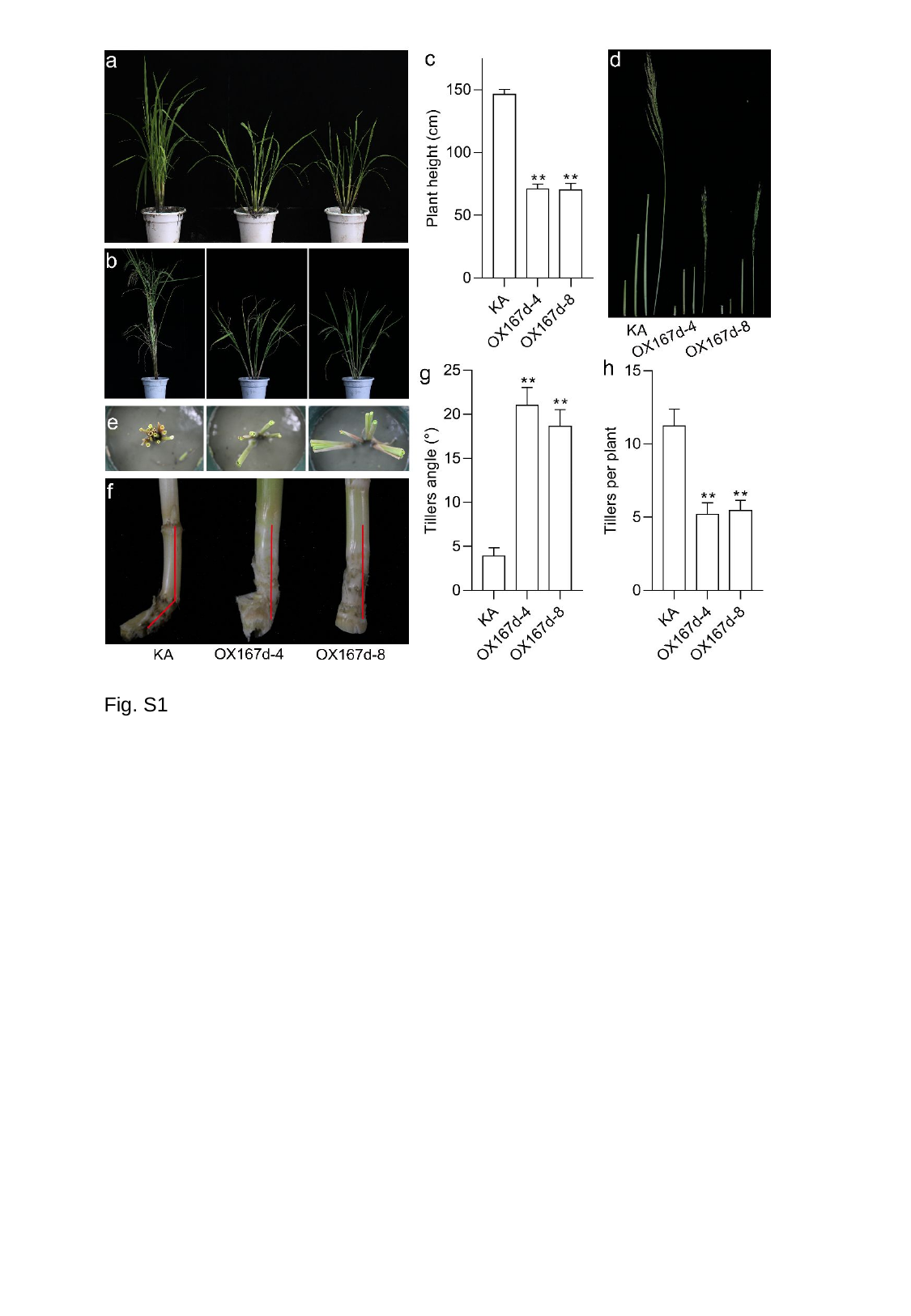

Fig. S1

## Slide 2
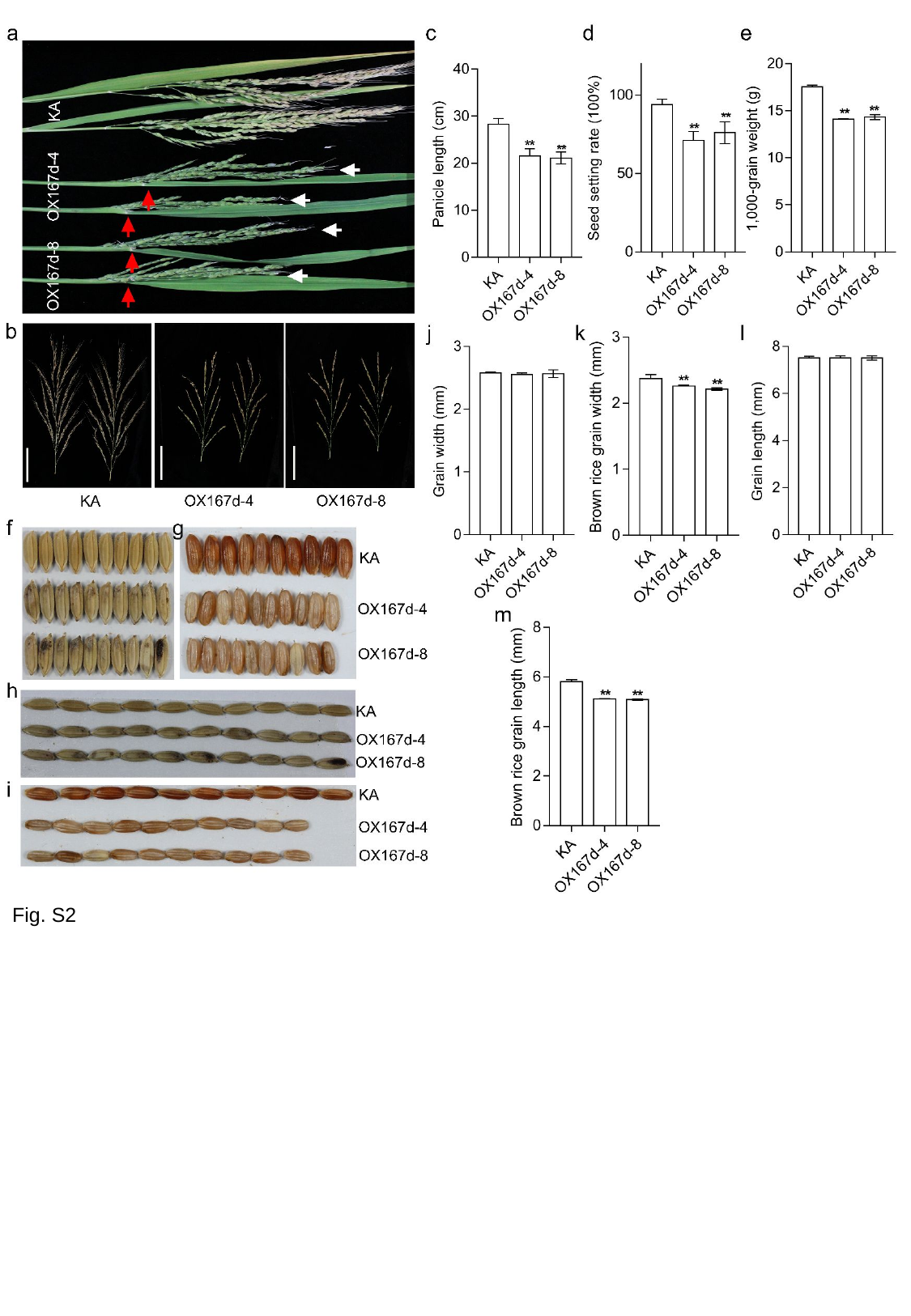

Fig. S2

## Slide 3
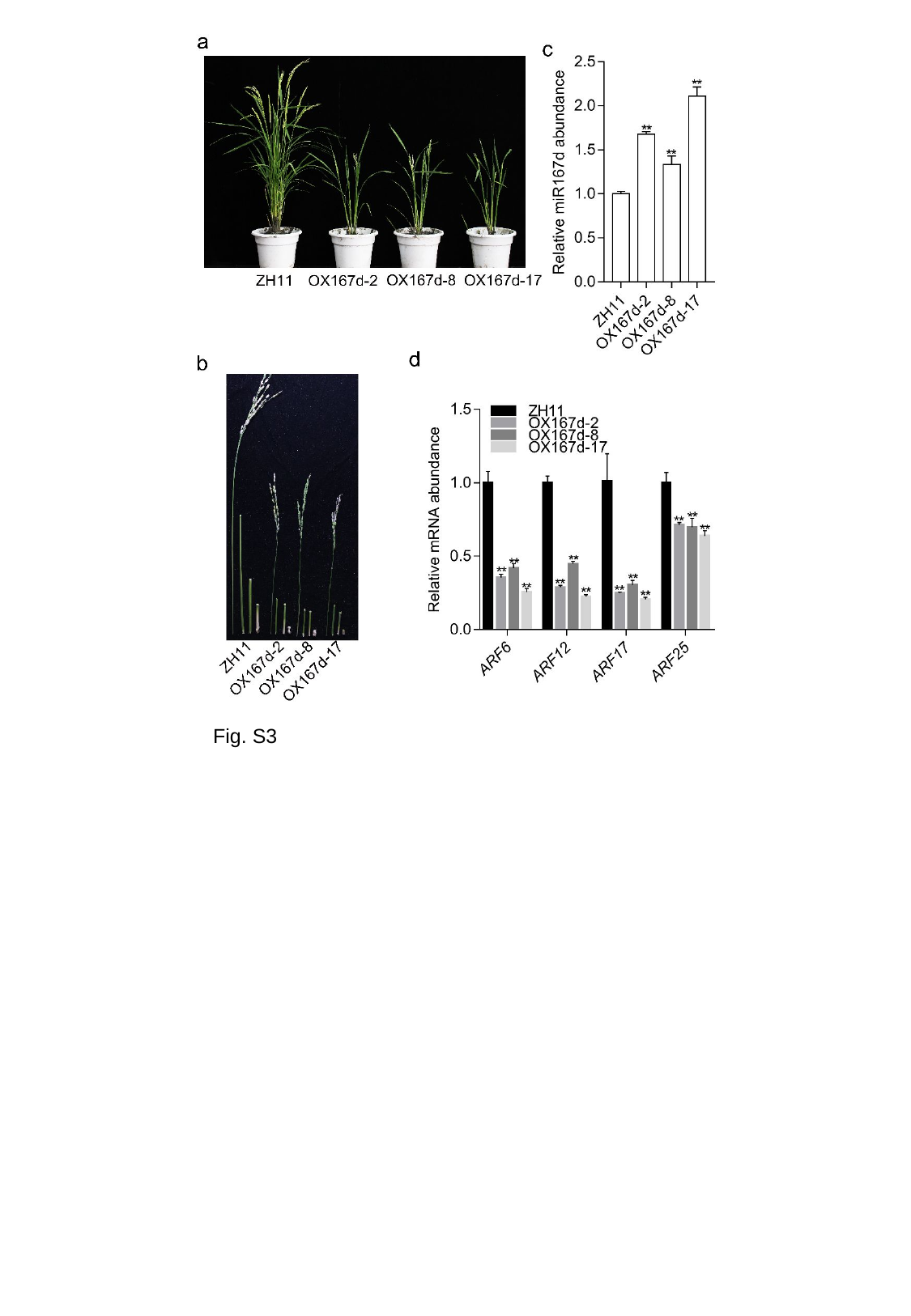

Fig. S3

## Slide 4
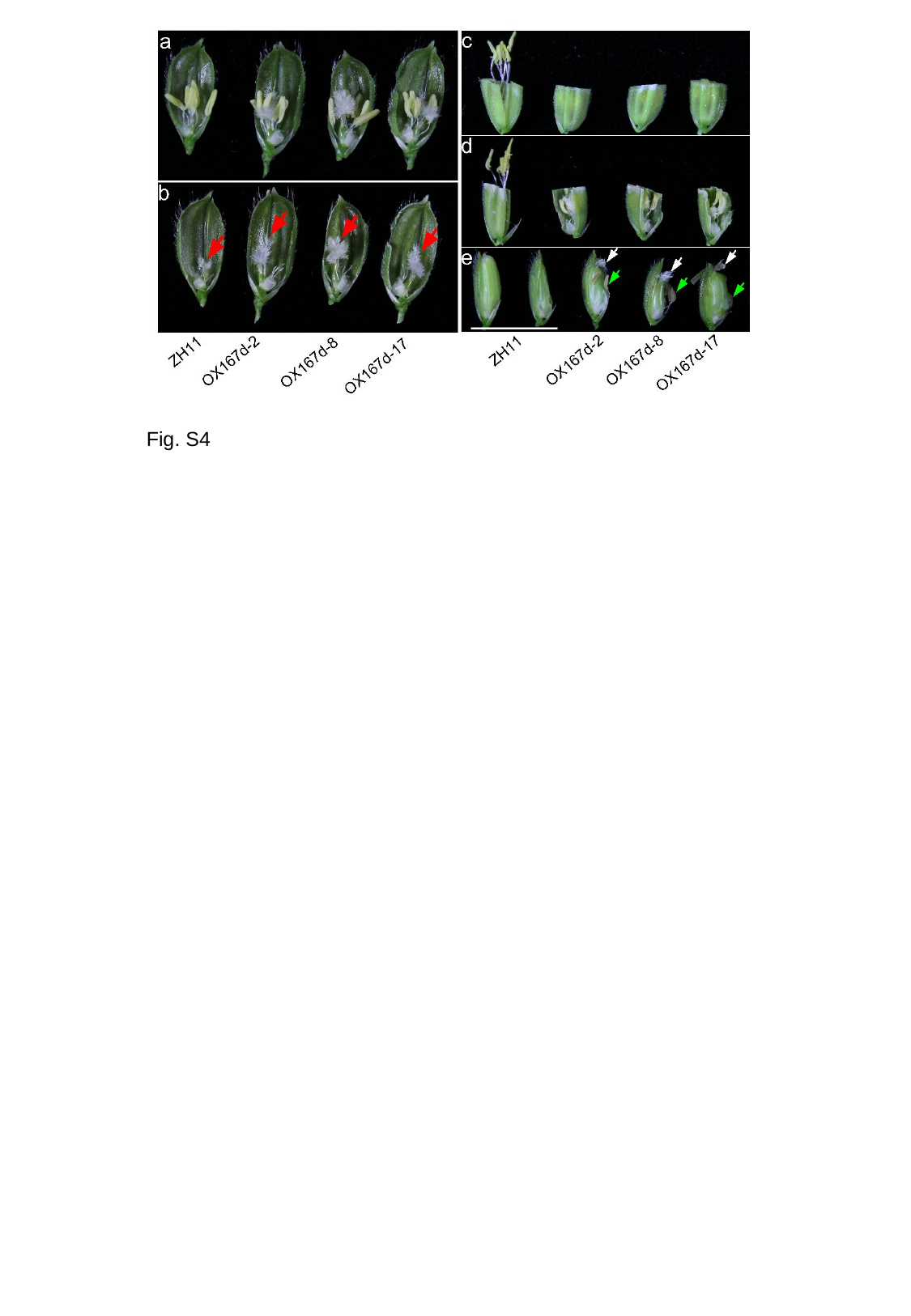

Fig. S4

## Slide 5
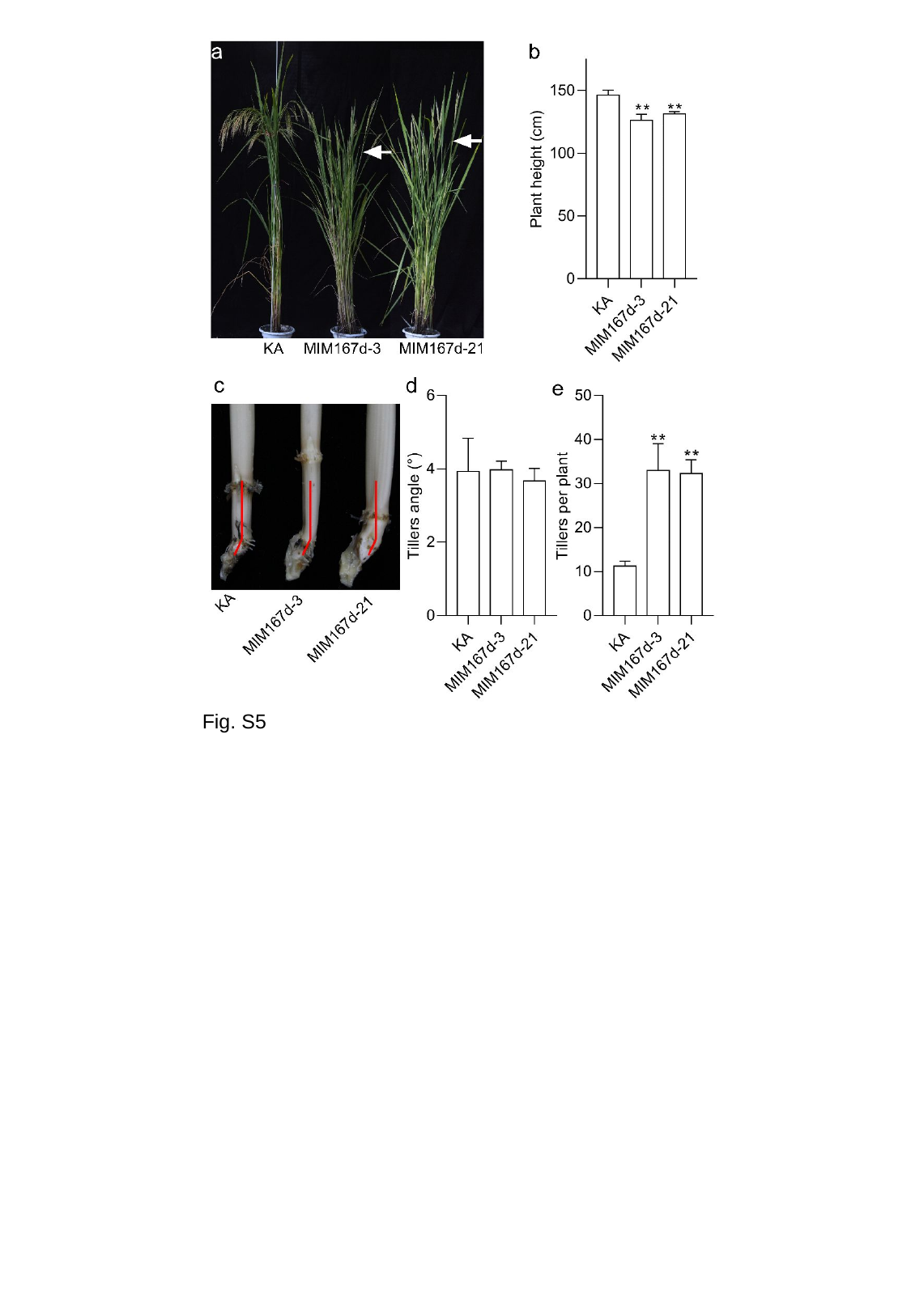

Fig. S5

## Slide 6
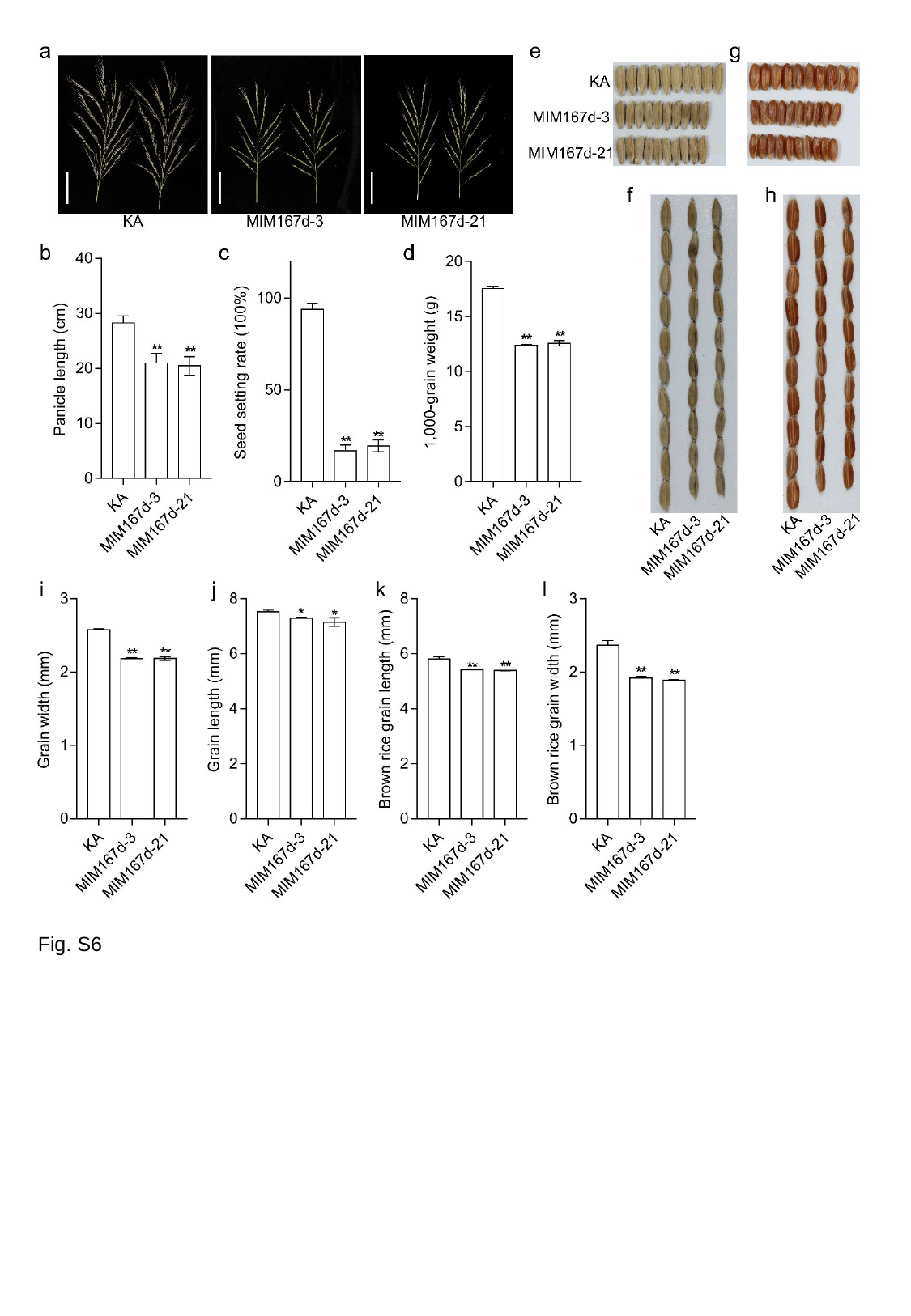

Fig. S6

## Slide 7
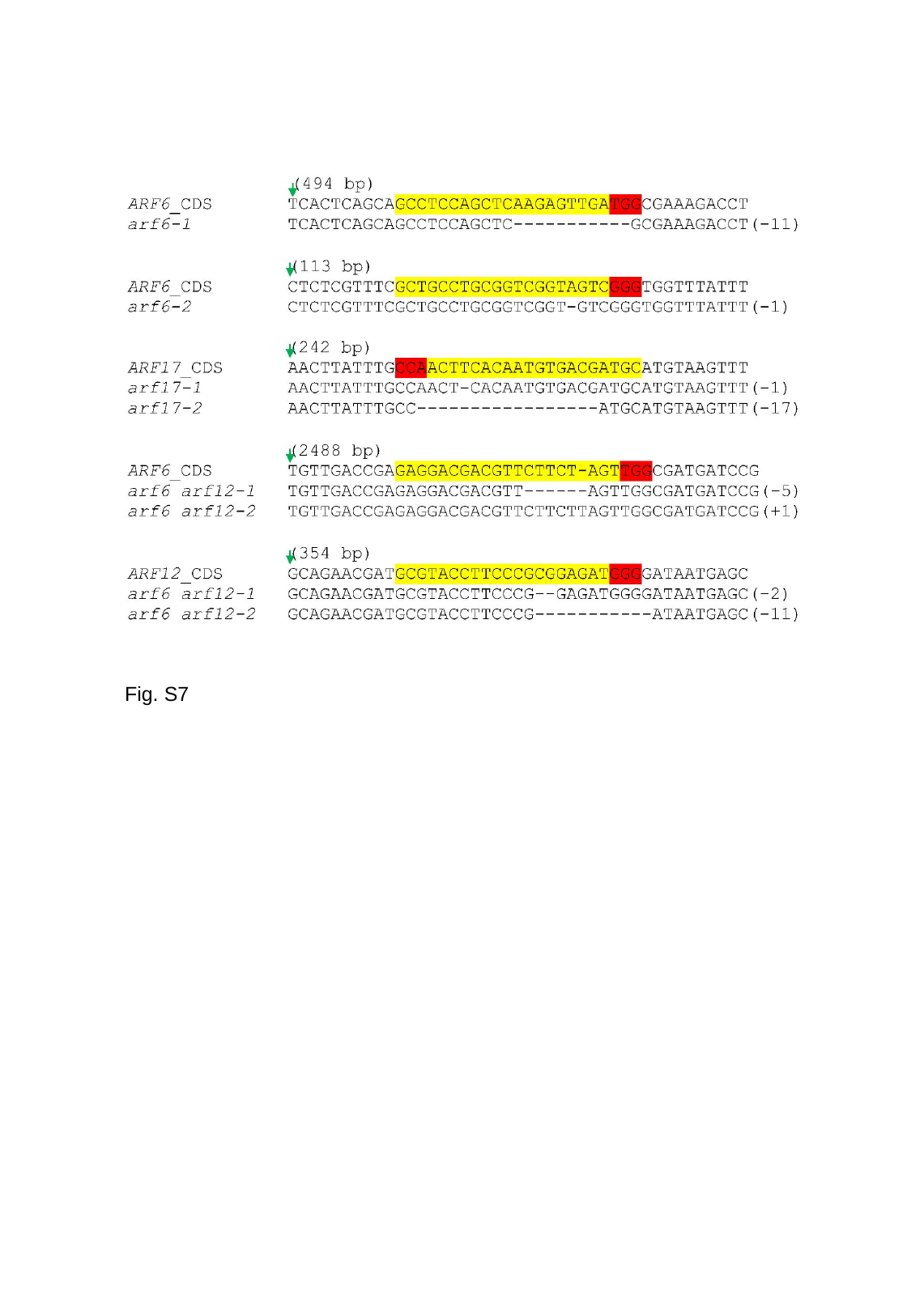

Fig. S7

## Slide 8
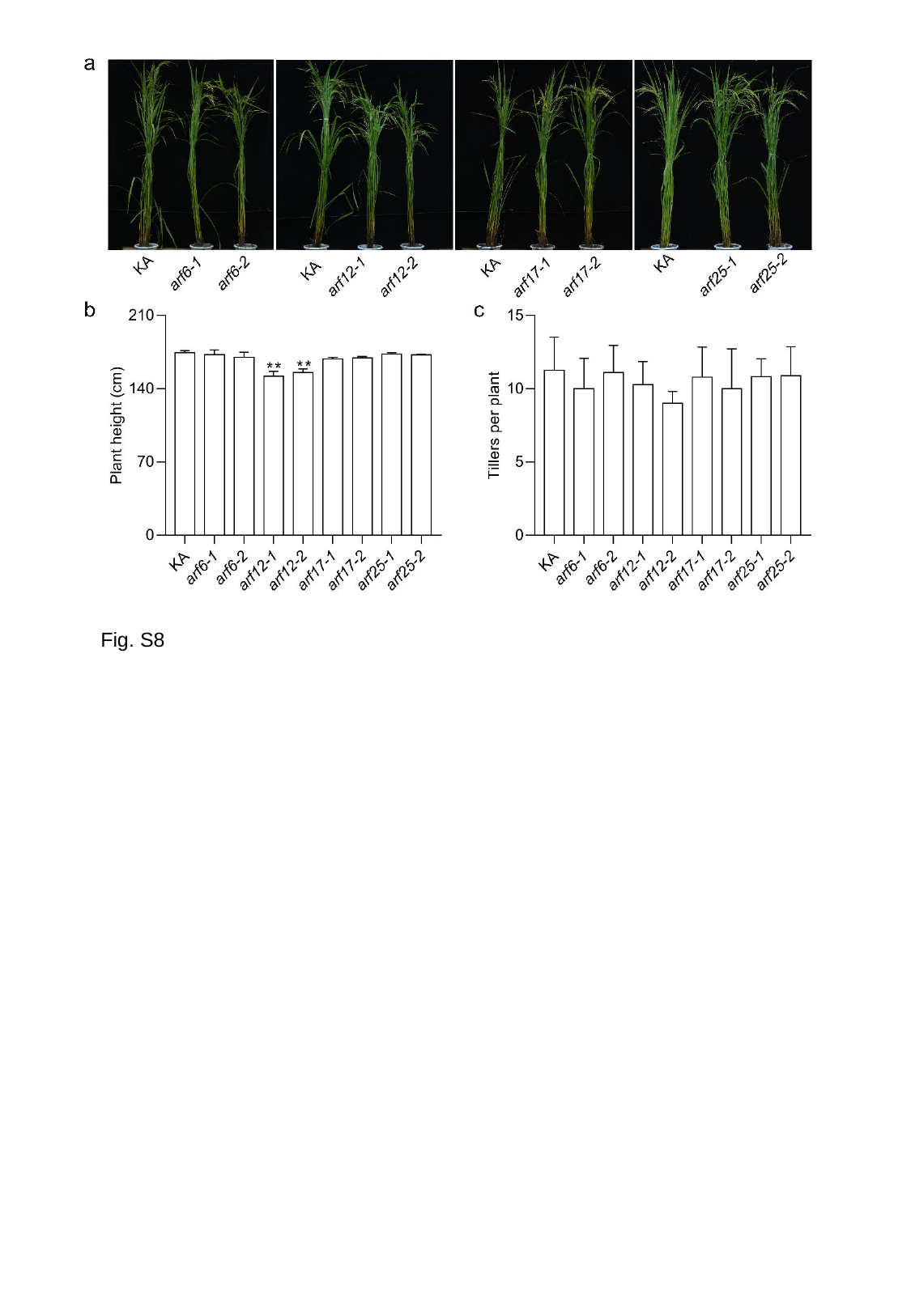

Fig. S8

## Slide 9
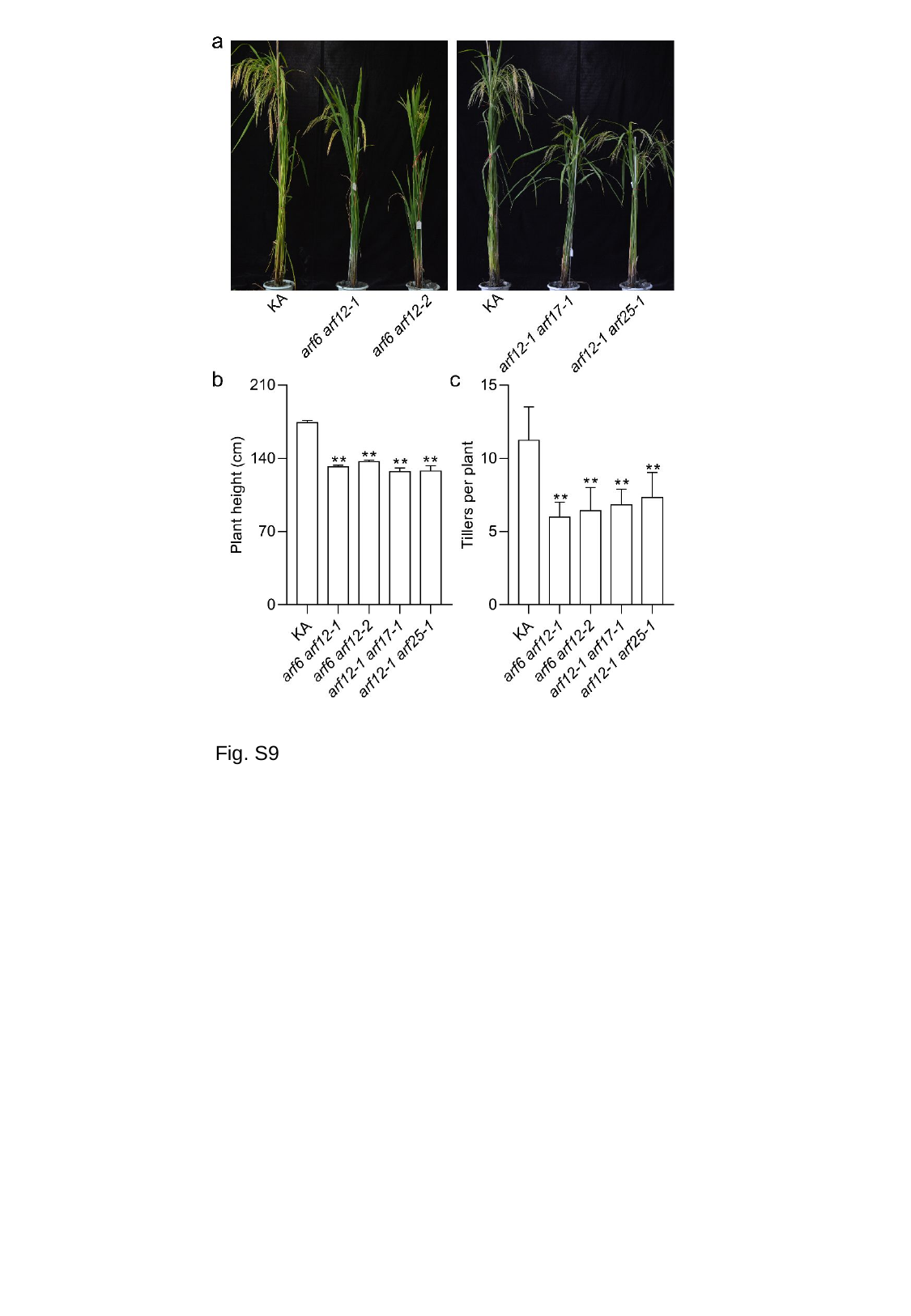

Fig. S9
